# The Many Faces of Dengue Infection: Dengue Infection Masquerading as Acute Airway Edema

**DOI:** 10.7759/cureus.115279

**Published:** 2026-08-27

**Authors:** Arkaprava Singharoy, Payel Bose, Tanmay Banerjee, Sagnik Das, Prerona Bhowmick

**Affiliations:** 1 Critical Care Medicine, Manipal Hospital, EM Bypass, Kolkata, IND; 2 Critical Care Medicine, Manipal Hospital, Siliguri, IND

**Keywords:** airway obstruction, angioedema, critical care, dengue, supraglottic edema

## Abstract

Acute supraglottic edema is an uncommon but potentially fatal cause of upper-airway obstruction. Although dengue infection is associated with diverse systemic manifestations, isolated upper-airway involvement is exceedingly rare and may pose a significant diagnostic challenge. We report an elderly woman who initially presented with features of acute stroke but was subsequently found to have life-threatening airway edema as a manifestation of acute dengue infection. Neuroimaging excluded acute cerebrovascular disease. Within six hours, she rapidly developed inspiratory stridor, hypoxemia, and hypercapnic respiratory failure requiring emergency endotracheal intubation. Flexible fiberoptic laryngoscopy demonstrated diffuse supraglottic and laryngopharyngeal edema, leading to compromised airway. Initial blood investigations revealed thrombocytopenia and a positive dengue NS1 antigen (sent in view of the ongoing dengue endemic), establishing the diagnosis of acute dengue infection. Conservative management comprising mechanical ventilation, corticosteroids, antibiotics, electrolyte correction, and serial fiberoptic airway assessment resulted in complete recovery without tracheostomy. This case highlights the evolving diversity of presenting clinical features associated with dengue infections. The pathophysiology of isolated airway edema in dengue is quite unknown and is beyond the scope of this article, but prompt pharmacological management, along with airway protection, reflects the reversible nature of this symptom and the likelihood of an underlying non-IgE-mediated hypersensitivity-related pathophysiological process. Early recognition of evolving airway compromise and prompt multidisciplinary intervention are essential for favorable outcomes.

## Introduction

Dengue is one of the most common arboviral infections worldwide and represents a major public health problem in tropical and subtropical countries. Although the classical manifestations include fever, malaise, thrombocytopenia, capillary leakage, and hemorrhagic complications, an increasing number of atypical presentations involving the neurological, cardiac, hepatic, renal, and respiratory systems have been recognized. Nevertheless, otorhinolaryngological manifestations remain distinctly uncommon, particularly those involving the upper airway [1].

Acute supraglottic edema is a life-threatening emergency that may rapidly progress to complete airway obstruction if not recognized promptly. In adults, the differential diagnosis includes infectious supraglottitis, medication-induced angioedema, allergic reactions, autoimmune disorders, trauma, and less commonly systemic inflammatory diseases. Early airway protection is the cornerstone of management because delayed intervention may result in catastrophic airway loss [2-5].

Acute stroke remains the most frequent diagnostic consideration in elderly patients presenting with sudden-onset dysarthria. However, progressive stridor, dysphonia, or unexplained hypoxemia should immediately prompt reassessment for airway pathology [2,3].

This case report highlights an atypical presentation of possible dengue fever in which life-threatening supraglottic edema requiring emergency airway management occurred as the only initial symptom, without fever or constitutional symptoms, in an elderly woman, emphasizing the need for greater clinical vigilance and early laboratory confirmation in endemic regions. In severe dengue, increased vascular permeability due to endothelial dysfunction can result in plasma leakage into the extravascular compartment, leading to pleural effusions, ascites, hemoconcentration, and, in severe cases, circulatory compromise [6]. Since dengue fever can lead to serious complications such as dengue hemorrhagic fever and shock syndrome, early detection of atypical symptoms is critical for appropriate interventions [1].

## Case presentation

An 84-year-old woman presented to the emergency department of our hospital in West Bengal, India, with sudden-onset slurring of speech, imbalance while walking, and one episode of vomiting approximately four hours before admission. She had experienced mild gait imbalance for nearly one year, which had partially improved with physiotherapy, but denied any previous episodes of dysphagia, stridor, allergic reactions, facial swelling, or airway compromise. Her medical history included hypertension, dyslipidemia, vitamin D deficiency, grade I fatty liver, and bilateral cataract surgery. Her regular medications included cilnidipine-based antihypertensive therapy, atorvastatin-clopidogrel, calcium supplementation, and vitamin D. There was no known history of medication allergy or recent initiation of any new medication.

On arrival, she was conscious with mild dysarthria but had no definite focal neurological deficits. Initial hemodynamic parameters were stable. Initial vital signs and laboratory investigations are summarized in Table 1.

**Table 1:** Initial Vital Signs and Laboratory Investigations on Admission Abbreviations: SpO₂, peripheral oxygen saturation; CRP, C-reactive protein; hs-cTnI, high-sensitivity cardiac troponin-I; NS1, non-structural protein 1; HPF, high-power field.

Parameter	Patient Value	Reference Range
Temperature	36.8 °C	36.5–37.5 °C
Heart rate	86 beats/min	60–100 beats/min
Blood pressure	132/78 mmHg	90–140/60–90 mmHg
Respiratory rate	18 breaths/min	12–20 breaths/min
Oxygen saturation (SpO₂)	97% (room air)	≥95%
Hemoglobin	11.8 g/dL	12.0–15.0 g/dL
Total leukocyte count	7.6 × 10⁹/L	4.0–11.0 × 10⁹/L
Platelet count	82 × 10⁹/L	150–450 × 10⁹/L
Serum sodium	129 mmol/L	135–145 mmol/L
Serum potassium	2.9 mmol/L	3.5–5.0 mmol/L
Blood urea	28 mg/dL	15–40 mg/dL
Serum creatinine	0.8 mg/dL	0.6–1.2 mg/dL
C-reactive protein (CRP)	70 mg/L	<5 mg/L
High-sensitivity cardiac troponin-I (hs-cTnI)	26 ng/L	<14 ng/L
Dengue NS1 antigen	Positive	Negative
Urine white blood cells (pus cells)	15–20/HPF	0–5/HPF

The patient had thrombocytopenia, hyponatremia (129 mmol/L), hypokalemia (2.9 mmol/L), elevated C-reactive protein, mild elevation of high-sensitivity cardiac troponin-I, and pyuria suggestive of urinary tract infection (Table 1). Electrocardiography showed no acute ischemic changes. Non-contrast computed tomography (CT) of the brain demonstrated no acute intracranial hemorrhage or infarction. The initial differential diagnosis included acute cerebrovascular event versus metabolic encephalopathy secondary to dyselectrolytemia. Appropriate neurological evaluation was undertaken while correction of electrolyte abnormalities and supportive therapy were initiated.

**Figure 1:**
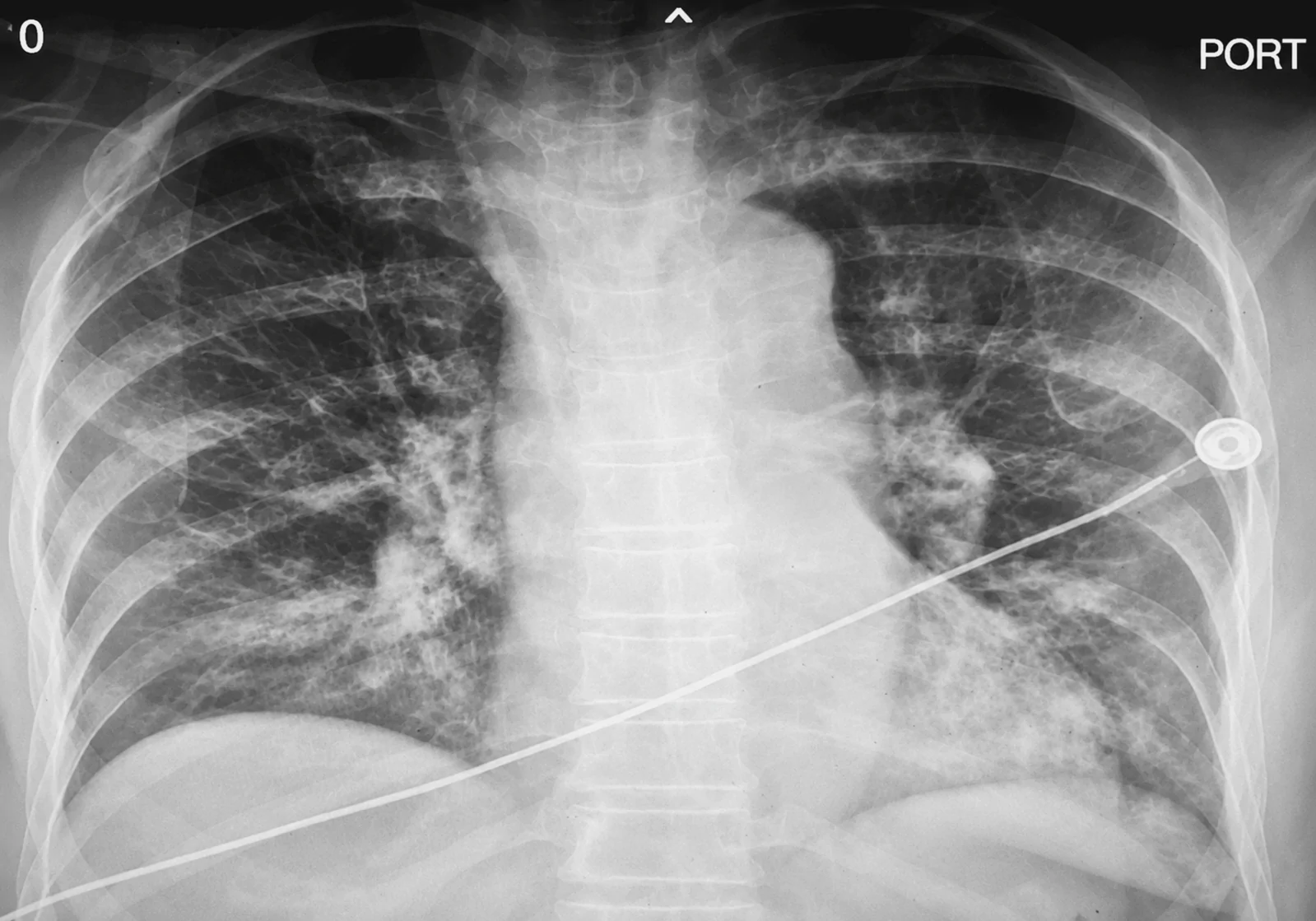
Chest radiograph on the day of admission (D1) showing no pleural effusion or radiographic evidence of capillary leak.

Following this, within a few hours, she developed progressive inspiratory stridor, tachypnea, increased work of breathing, and hypoxemia with an increasing oxygen requirement. Arterial blood gas analysis at this stage showed a pH of 7.25, PaCO₂ of 58 mmHg, PaO₂ of 62 mmHg, and HCO₃⁻ of 25 mmol/L, consistent with acute hypercapnic respiratory failure with hypoxemia. In view of the impending airway compromise, emergency endotracheal intubation was performed using rapid-sequence induction. Direct laryngoscopy revealed marked edema involving the supraglottic structures with narrowing of the upper airway. The patient was transferred to the intensive care unit (ICU) and started on mechanical ventilation with sedation, corticosteroids, bronchodilator nebulization, enteral nutrition, and supportive intensive care. A urine culture was obtained in view of pyuria, while serial arterial blood gas analyses guided ventilatory management. The urine culture grew *Escherichia coli*, for which the patient was treated with appropriate antibiotics for a urinary tract infection. A chest X-ray was done, which showed no effusion or capillary leak (Figure 1).

In view of thrombocytopenia and the ongoing dengue endemic, dengue NS1 antigen testing was performed, which returned positive.

**Figure 2:**
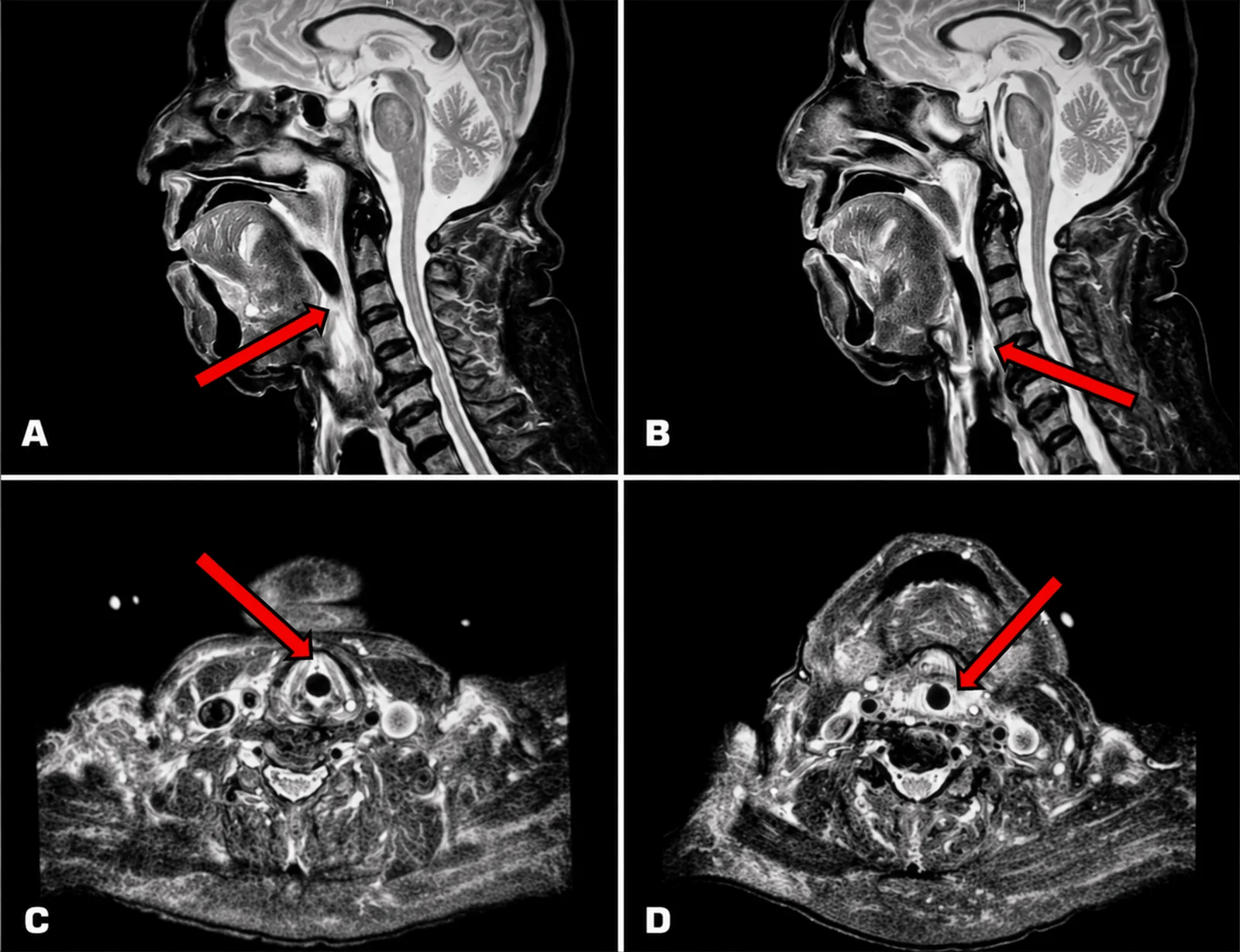
Magnetic resonance imaging of the brain and cervical region showed upper-airway edema with no evidence of acute ischemic stroke. (A, B) Sagittal T2-weighted images demonstrating diffuse upper-airway edema along the posterior pharyngeal wall and supraglottic vestibule (arrows). (C, D) Axial T2-weighted images at the level of the upper larynx showing circumferential edema of the aryepiglottic folds and pre-epiglottic fat spaces (arrows), resulting in critical narrowing of the residual airway passage.

Further evaluation was undertaken to determine the etiology of the upper-airway obstruction. Magnetic resonance imaging of the brain and cervical region showed upper-airway edema with no evidence of acute ischemic stroke (Figure 2).

**Figure 3:**
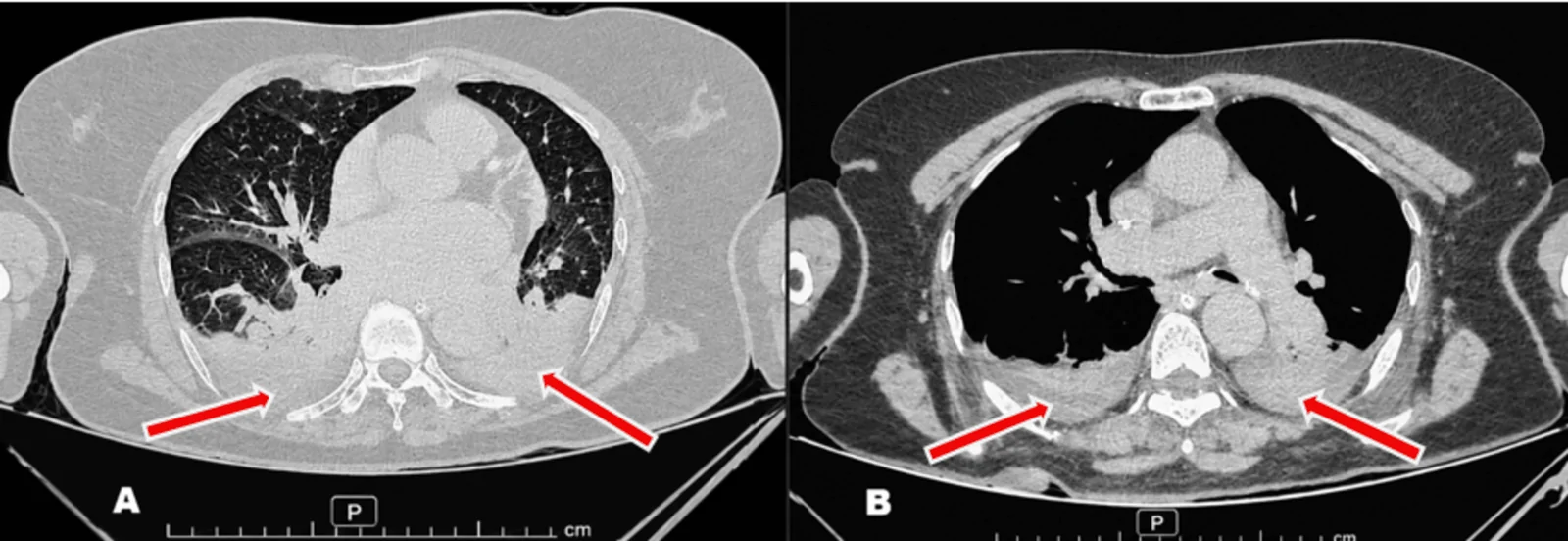
Computed tomography (CT) of the thorax on day 2 post-admission demonstrating pulmonary and mediastinal findings. (A) Lung window image showing mild bilateral pleural effusions with dependent bibasal atelectatic-consolidative changes (arrows). (B) Axial CT image in the mediastinal window demonstrating no evidence of tracheal compression, airway narrowing, or other mediastinal pathology (arrows indicate the pleural effusions).

Transthoracic echocardiography demonstrated preserved left ventricular systolic function (ejection fraction approximately 60%) with Grade I diastolic dysfunction and no regional wall motion abnormality. In view of mildly diminished breath sounds and a small rise in oxygen requirement, CT of the thorax demonstrated mild bilateral pleural effusions with dependent bibasal atelectatic-consolidative changes (Figure 3) without evidence of tracheal compression or mediastinal pathology. Flexible fiberoptic laryngoscopy performed by the otorhinolaryngology team demonstrated diffuse laryngopharyngeal and supraglottic edema with edematous arytenoid and pyriform regions, while the true vocal cords remained relatively preserved. No foreign body, abscess, or neoplastic lesion was identified. Infectious supraglottitis, bradykinin-mediated angioedema, and inflammatory upper-airway edema were considered in the differential diagnosis. Complement C4 and serum immunoglobulin E levels were measured to evaluate alternative causes of angioedema, and all other differentials (Table 2) were ruled out.

**Table 2:** Differential Diagnosis

Diagnosis	Supporting Features	Findings Against
Acute ischemic stroke	Sudden dysarthria, gait instability	CT and MRI negative
Infectious supraglottitis	Elevated CRP, respiratory infection	No abscess or epiglottic collection
Drug-induced angioedema	History of chronic antihypertensive therapy	No recent drug initiation or dose escalation
Bradykinin-mediated angioedema	Isolated upper-airway edema	Complement C4 levels within normal limits
Dengue-associated edema	NS1 positivity, thrombocytopenia, subsequent pleural effusion	Alternative etiology less likely

Apart from thrombocytopenia and mild bilateral pleural effusions, no other classical clinical manifestations of dengue were observed during the hospital course. The patient remained afebrile and had no documented rash, bleeding manifestations, ascites, or generalized edema. There was no clinical evidence of hepatic or renal injury, and she remained hemodynamically stable without shock or requirement for vasopressor support. Analgosedation was gradually tapered, following which the patient remained neurologically intact without focal deficits. Daily physiotherapy, spontaneous awakening trials, spontaneous breathing trials, and meticulous airway care were continued throughout the ICU stay. Serial flexible fiberoptic laryngoscopic examinations were performed to assess the progression and subsequent resolution of supraglottic edema and to guide the timing of extubation. Following demonstration of an adequate cuff leak and successful spontaneous breathing trial, the patient was successfully extubated on day six of hospitalization.

Electrolyte abnormalities normalized with appropriate replacement therapy. The patient remained clinically stable, with resolution of respiratory symptoms, and was subsequently discharged from the ICU. A summary of the patient’s hospital course is provided in Table 3.

**Table 3:** Timing of Clinical Course

Hospital Day	Clinical Events
Day 1	Sudden dysarthria, vomiting; suspected acute stroke
Day 1	CT brain negative for acute hemorrhage
Day 1	Rapid-onset stridor and respiratory failure; emergency intubation; thrombocytopenia; dengue NS1 positive
Day 1	MRI brain excluded acute infarction; fiberoptic laryngoscopy showed supraglottic edema
Day 2–3	Mechanical ventilation, steroids, antibiotics, electrolyte correction
Day 3–5	Serial fiberoptic examinations
Day 6	Resolution of edema; successful extubation
Day 7	Transfer from ICU

## Discussion

Dengue infection is mostly known to be a self-limiting febrile illness, with a subset of patients progressing to a severe form, characterized by plasma leakage and organ involvement. Following an incubation period of 3-7 days, patients enter a febrile phase, after which some progress to a critical phase of 24-48 hours, characterized by transient endothelial dysfunction with increased vascular permeability, resulting in plasma leakage and third spacing of fluid into the interstitial and serosal compartments. Current evidence suggests that this vascular leak is mediated by a complex interplay of host inflammatory responses, including pro-inflammatory cytokines such as tumor necrosis factor-α (TNF-α), mast cell-derived vasoactive mediators, platelet-activating factor, angiopoietin imbalance, and disruption of the endothelial glycocalyx by dengue non-structural protein-1 (NS1). Clinically, the vascular leak manifests as pleural effusions, ascites, gallbladder wall edema, and, in severe cases, circulatory shock [6]. Published reports have documented laryngeal edema predominantly in patients with established severe dengue, where it has been attributed to capillary leak compounded by aggressive fluid resuscitation [7]. Although atypical manifestations of dengue are increasingly recognized, involvement of the upper airway has rarely been reported [8]. Rare cases of laryngeal or vocal cord hematoma secondary to dengue-associated thrombocytopenia have also been reported [7,9]. The reported cases and the present case are summarized in Table 4.

**Table 4:** Previously Reported Upper-Airway Manifestations Associated With Dengue Infection and Comparison With the Present Case

Study	Patient Characteristics	Dengue Severity/Context	Upper-Airway Manifestation	Airway Intervention/Outcome	Proposed Mechanism
Saran & Azim, 2017 [7]	20-year-old woman	Severe dengue with respiratory failure, shock, and marked thrombocytopenia	Bilateral arytenoid swelling/laryngeal edema with recurrent post-extubation stridor	Repeated re-intubation followed by tracheostomy; subsequently decannulated and discharged	Capillary leakage with possible contribution from fluid resuscitation
Tamilarasan et al., 2018 [9]	Patient with dengue fever and severe thrombocytopenia	Dengue-associated thrombocytopenia	Vocal cord hematoma	Conservative management; complete recovery	Thrombocytopenia-associated bleeding
Current case	84-year-old woman	Acute dengue with thrombocytopenia and subsequent bilateral pleural effusions	Diffuse supraglottic and laryngopharyngeal edema, involving the arytenoid and pyriform regions, with relatively preserved true vocal cords	Emergency endotracheal intubation followed by serial fiberoptic laryngoscopy; successfully extubated on day 6	Possible dengue-associated plasma leakage involving the upper airway; alternative causes were evaluated and excluded

Thus, this case is unique for the rarity of acute airway edema as a primary sign of dengue fever, which is rarely reported in the medical literature. This uncommon presentation poses a diagnostic challenge since clinically acute supraglottic edema is usually not associated with dengue virus infection. The patient's initial symptoms of dysarthria, gait instability, and vomiting appropriately prompted activation of an acute stroke evaluation pathway. Only after rapid progression to inspiratory stridor and respiratory failure did the primary diagnosis shift toward an upper-airway emergency. This emphasizes the importance of repeated bedside reassessment in patients with evolving symptoms rather than reliance on the initial working diagnosis [2,3].

Acute dengue infection was confirmed by NS1 antigen positivity, thrombocytopenia, and later development of bilateral pleural effusions, which demonstrated evidence of systemic plasma leakage. This temporal sequence raises the possibility that the supraglottic tissues represented the earliest clinically apparent site of dengue-associated capillary leak or a non-IgE-mediated hypersensitivity reaction or complement activation, while other differentials of supraglottic edema, such as infectious supraglottitis, drug-induced and IgE-mediated angioedema, and structural causes, ruled out [2-5]. The fact that the airway edema subsided with time and steroid therapy might be an indication towards an immune-mediated causative mechanism.

The absence of fever, malaise, headache, or any other constitutional symptom of dengue illness further hindered the onset of clinical suspicion of the disease, even though afebrile dengue has been described in the literature [10]. Therefore, in dengue-endemic regions, acute dengue infection may be considered in the differential diagnosis of otherwise unexplained supraglottic edema or signs of plasma leakage after an extensive evaluation, even when the classic symptoms of dengue are absent.

This case report should be treated as an index of the evolving nature of dengue illness symptoms and should encourage research involving larger patient cohorts to correlate dengue virus infection with upper-airway edema and identify its precise pathophysiological pathways.

## Conclusions

This case describes life-threatening supraglottic edema requiring emergency airway management as an exceptionally rare presentation of acute dengue infection, in the absence of fever, myalgia, and other classical manifestations of dengue. The case highlights that dengue-associated upper-airway edema may improve with glucocorticoid therapy, although the underlying mechanism remains uncertain. Early airway protection, repeated clinical reassessment, timely dengue testing in endemic settings, and targeted pharmacotherapy were central to the successful outcome. Upper-airway edema may be recognized as a rare but potentially life-threatening manifestation of dengue infection. In endemic regions, unexplained supraglottic edema or rapidly progressive stridor should prompt consideration of dengue even when fever is absent.
